# Unusual Localisation for Onychomatricoma on the 5th Toenail: A Case Report and Review of the Literature

**DOI:** 10.1155/2016/1853495

**Published:** 2016-07-13

**Authors:** A. Coutellier, I. Théate, O. Vanhooteghem

**Affiliations:** ^1^Department of Dermatology, Sainte Elisabeth Hospital, 5000 Namur, Belgium; ^2^Department of Pathology, Institute of Pathology and Genetics, 6041 Gosselies, Belgium

## Abstract

Onychomatricoma is a rare and benign tumour of the nail matrix but originates rarely from the ventral portion of the proximal nail fold. This tumour is characterised by fingerlike projections that invade the nail plate. This lesion, of unknown aetiology, is typically asymptomatic with slow progression. Localisation on the finger is the most frequently described. We report the case of a 68-year-old woman who has an onychomatricoma in an unusual location, the fifth toe of the left foot. Due to its clinical appearance, the tumour can be confused with and treated as onychomycosis. However, if it is resistant to an oral antifungal well behaved treatment, one must consider onychomatricoma diagnosis.

## 1. Case Report

**Figure 1:**
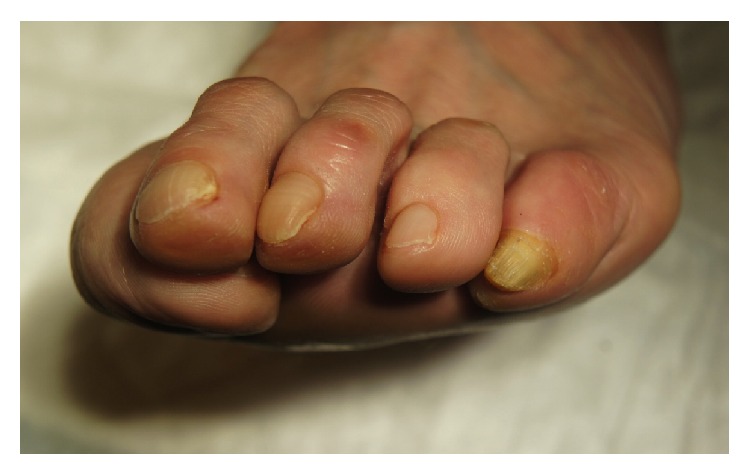
Pachyonychia and xanthonychia on the fifth toenail.

**Figure 2:**
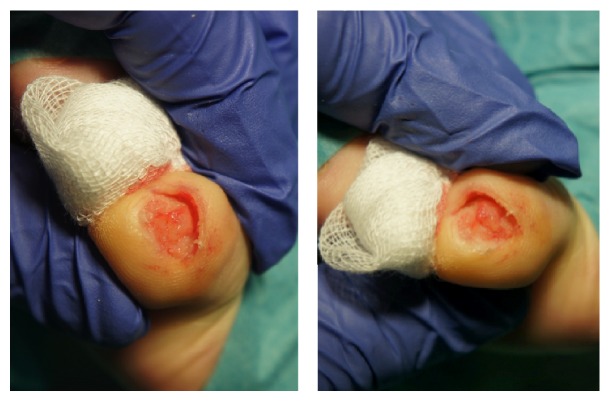
The nail avulsion exposes a villous tumour.

A 68-year-old woman went to a dermatologist for an opinion regarding the thickening and yellowing of the nail plate on the fifth toe of the left foot, which had appeared gradually over the past 2 years. She had no history of prior traumatism, and the lesion was pain-free. Clinical examination showed a thickening of the nail plate, a xanthonychia, and distal splinter haemorrhages of the nail plate on the 5th toe (Figure 1). Dermoscopy revealed visible holes in the thick portion of the nail plate. All other nails on both the hands and the feet were lesion-free. She had no relevant medical history. Analysis of two mycological samples was negative. For diagnostic purposes, a nail avulsion under local anaesthesia was performed, which revealed the presence of a villous tumour on the nail matrix (Figure 2). The tumour was excised, and after eight months of follow-up, the nail regrew without any signs of relapse or ungual dystrophy.

## 2. Discussion

Onychomatricoma (OM) is a rare and benign fibroepithelial tumour of the nail matrix. It was first described in 1992 by Baran and Kint. They have reported three cases of filamentous funnel-shaped tumours in the nail matrix [1]. To date, there have been less than eighty cases reported in the literature. In most cases, it was found in Caucasian adults approximately fifty years of age, while only few cases have been reported in Black patients. One case was reported in a child [2, 3]. The sex ratio was two females to one male [4]. In 75 percent of cases [4–6], the localisation is in the fingers, and the middle finger is affected in two of three cases [7]. When the OM reaches toenails, most cases are found in the big toe and sometimes in the second or the third toe [4]. In a study about thirty OM patients, eleven cases were described on the toenails: seven in the big toenail, two in the second toe, and two in the third toe [4]. To our knowledge, up to now, no case has been reported on the fifth toenail.

**Figure 3:**
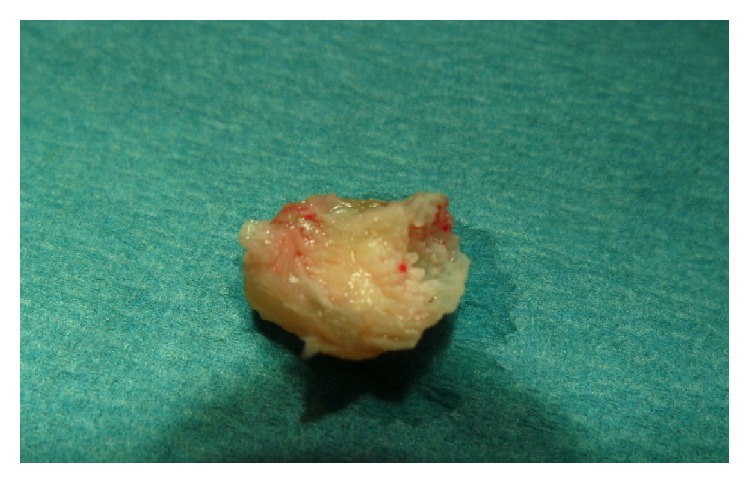
Cavitation in the nail plate occupied by the fingerlike projections of the matrix villous tumour.

**Figure 4:**
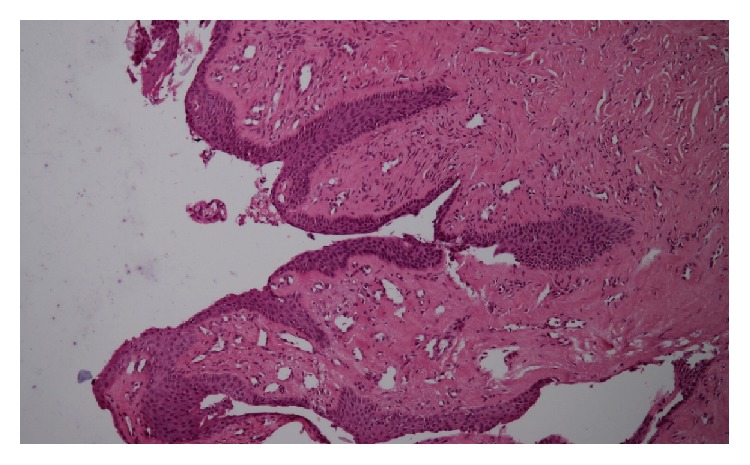
Histology: papillary projections “fingered gloves” with a matrix-type epithelium devoid of a granular layer.

It is a rare tumour for which nail plate modification originates in the matrix [5, 8]. Occasionally, the tumour originates from the ventral part of the proximal nail fold. The cause of onychomatricoma remains unknown. Traumatic or microtraumatic factors have been suggested, but these have not been confirmed [5]. An aberrant expression of B-cadherin may be involved in the pathogenesis of OM [9]. Genetic alterations in the 11th chromosome with overexpression of STIM-1 and cathepsin C may also be implicated [5]. Asymptomatic tumours have a slow growth. The typical clinical presentation combines the following five main indications: thickening of the nail, longitudinal xanthonychia, longitudinal and transverse overcurvature of the nail plate, distal and mainly splinter haemorrhages, and multiple perforations on the free edge of the thickened nail plate. A nodular tumefaction at the base of the nail's alteration and bleeding are also associated with this tumour [7, 10]. Some variants of OM are described as follows: giant forms, pigmented forms associated with a longitudinal melanonychia, OM associated with a dorsal pterygium, or an onychomycosis [5–7, 11]. The tumour is frequently diagnosed and treated as onychomycosis [8]. OM is a predisposing factor for onychomycosis, and they are often associated. But unusual resistance to oral antifungal well behaved treatment should sometimes warrant considering underlying OM. The diagnosis of OM is based on the classic clinical signs previously mentioned. Additional examinations, such as dermoscopy, ultrasound, magnetic resonance imaging, and confocal microscopy, may aid in its diagnosis. The dermoscopic examination allows for better visualisation of the presence of the perforations on the free edge of the thickened nail as well as haemorrhagic streaks and white longitudinal ridges. Using high-resolution ultrasound, the tumour appears hypoechoic next to the matrix and on the fingerlike projections. The magnetic resonance imaging (with digital antenna) shows the fingerlike projections of the nail matrix on sagittal sections [7, 8]. The treatment consists of a complete surgical excision of the tumour. The avulsion of the nail plate under local anaesthetics reveals a matrix villous tumour that resembles a “sea anemone” with its characteristic digitations, in a thickened bored nail with multiple cavities and extending into the proximal nail (Figure 3). These digitations are responsible for the thickening of the nail [7]. The “fingerlike” projections of the tumour, which emerge from the matrix, are resected. Three distinct histological characteristics define the OM [7–9, 12, 13]. The fibroepithelial tumour has two zones. The proximal portion (proximal periungual area) is characterised by deep vertical epithelial invaginations centred on empty V shaped cavities. The stroma ranges from being partially to highly cellularised and can be myxoid, fibromyxoid, or collagenous. The stroma is composed of fibroblasts, mast cells, and sometimes multinucleated giant cells. The distal portion of the lunula is comprised of multiple digitations set along connective tissues and lined with an epithelium without a granular layer (Figure 4). In addition, the matrix tumour contains two layers (superficial and deep). The stroma of the deep layer is less cellularised with denser collagen bundles. Finally, the villi are covered with matrix epithelium that has a keratogenous zone. Immunohistochemical analysis is usually not necessary; however, it facilitates the diagnosis because OM differs from other lesions by expressing CD34 but not CD99 [12, 14]. The nail regrowth is usually excellent. In rare cases, secondary ungual dystrophy has been observed depending on the degree of preservation of the nail matrix. Recurrences are very rare, some few cases observed when the excision was incomplete [5, 7, 12]. The Mohs micrographic surgical technique allows the removal of the entire lesion, preserving a maximum amount of healthy tissue [15].

## 3. Conclusion

Onychomatricoma is a rare benign tumour of the nail matrix commonly occurring on the fingernails. This case is of interest due to its unusual location on the fifth toenail.
